# The Effect of UV Irradiation on Vitamin D_2_ Content and Antioxidant and Antiglycation Activities of Mushrooms

**DOI:** 10.3390/foods9081087

**Published:** 2020-08-10

**Authors:** Francesca Gallotti, Vera Lavelli

**Affiliations:** Department of Food, Environmental and Nutritional Sciences (DeFENS), University of Milan, 20133 Milan, Italy; francesca.gallotti@unimi.it

**Keywords:** mushroom, vitamin D, antioxidant activity, reducing capacity, glycation

## Abstract

Mushroom irradiation has been considered a sustainable process to generate high amounts of vitamin D_2_ due to the role of this vitamin for human health and of the global concerns regarding its deficient or inadequate intake. Mushrooms are also receiving increasing interest due to their nutritional and medicinal properties. However, there is still a knowledge gap regarding the effect of UV irradiation on mushroom bioactive compounds. In this study, two of the most cultivated mushroom species worldwide, *Agaricus bisporus* and *Pleurotus ostreatus*, were irradiated with UV-B, and the effect of processing was investigated on the contents of vitamin D_2_ as well as on antioxidant and antiglycation activities. UV irradiation increased vitamin D_2_ up to 57 µg/g d.w, which is an adequate level for the fortification of a number of target foods. UV irradiation decreased the antioxidant activity when measured by the Folin–Ciocalteu reagent, the 2,2-diphenyl-1-(2,4,6 trinitrophenyl) hydrazyl radical assay and the ferric ion-reducing antioxidant power assay, but did not decrease the mushroom’s ability to inhibit glycation of a target protein. These results open up a new area of investigation aimed at selecting mushroom species with high nutraceutical benefits for irradiation in order to maintain their potential properties to inhibit oxidative and glycation processes responsible for human diseases.

## 1. Introduction

Deficient and inadequate vitamin D intake (vitamin D represents both D_2_ and D_3_ forms) is a worldwide public health issue causing both skeletal diseases and increased risk of various chronic diseases [1]. Sources of vitamin D for humans are sunlight exposure, yielding vitamin D_3_; a few foods of animal origin providing vitamin D_3_; and yeasts and mushrooms, providing vitamin D_2_. Both vitamin D_2_ and D_3_ are metabolized in the liver to 25-hydroxyvitamin D (25(OH)D) and then in the kidneys to 1,25-dihydroxyvitamin D (1,25(OH)2D). According to the serum concentration of 25(OH)D, vitamin D status is defined as deficient when serum 25(OH)D is lower than 25 nmol/L (10 ng/mL) and insufficient when serum 25(OH)D is between 25 and 50 nmol/L (10–20 ng/mL). Vitamin D status can also be assessed by validated food questionnaires [2]. Populations at risk of vitamin D deficiency are young children, the elderly, pregnant women and non-western immigrants in Europe [1]. The occurrence of vitamin D deficiency was found to be 48.1% in a Chinese population of preschool-aged children [3]. A large survey throughout Europe has shown that the intake of vitamin D is inadequate for 77–100% of adults (19–64 years old) and for 55–100% of elderly adults (>64 years old) [4]. From the 2005–2016 National Health and Nutrition Examination Surveys (NHANES), the prevalence of inadequacy for vitamin D intake among US population was found to be 95% [5].

Despite the fact that vitamin D_3_ can be formed by the conversion of its precursors in the skin upon sunlight exposure, the above-reported studies document that this process is not sufficient to ensure appropriate levels of this vitamin. Moreover, the intake of vitamin D_3_ from animal-based foods is not a proper solution because the current food systems, which are highly dependent on animal-based food sources, are not sustainable from an environmental point of view but also from a health and food security perspective [6]. Hence, one strategy to overcome the global demand of vitamin D in a sustainable way is to produce this vitamin from mushroom irradiation. This way, vitamin D can be delivered through fortified foods [7]. The amount of vitamin D_2_ in mushrooms is generally low. However, mushrooms have high amounts of the vitamin D_2_ precursor, i.e., ergosterol. Processes that generate vitamin D_2_ from ergosterol have been developed by UV irradiation on either the fresh or dried fruit body [8]. Recent trends have tested the efficiency of vitamin D_2_ generation by combining supercritical CO_2_ extraction with dissolution of the extract in ethanol or methanol followed by UV irradiation [9]. *Pleurotus* spp. are among the basidiomycetes that can be used for vitamin D_2_ generation because their cultivation can be performed on various agri-food waste at a low cost [10,11]. Another basidiomycete of interest is *Agaricus bisporus*, which is the most cultivated mushroom worldwide [12].

Mushrooms are known to have medicinal properties and interest in this food source is increasingly expanding due to many studies that demonstrated their potential roles on human health due to antioxidant, antitumor, antimicrobial, anti-inflammatory, immunomodulator, antiatherogenic and hypoglycemic activities [10,11].

There is still a knowledge gap regarding the effect of UV irradiation on mushrooms’ bioactive properties. Nevertheless, it is known that UV radiation can promote photo-oxidation via two major routes. The first of these involves direct photo-oxidation arising from the absorption of UV radiation by chromophores, thereby generating excited state species (singlet or triplets) or radicals as a result of photo-ionization. The second major process involves indirect oxidation of targets via the formation and subsequent reactions of singlet oxygen (^1^O_2_). Antioxidant enzymes that eliminate ^1^O_2_ have not evolved. Instead, the highly reactive ^1^O_2_ can interact with potential targets by either physical quenching or a chemical reaction. The former results in energy transfer and de-excitation of the singlet state but no chemical change in the energy acceptor. The latter causes modification of the target and in the initiation of radical type reactions [13]. Hence, it may be expected that UV irradiation affects the potential ability of mushrooms to prevent cell damage.

Oxidative stress has been found to mediate cell damage, thus triggering a number of human diseases [14]. The term is used to describe the condition of oxidative damage resulting when the critical balance between reactive oxygen species (ROS) enzymatic and non-enzymatic generation and antioxidant defenses (low molecular weight antioxidants, antioxidant enzymes and repair enzymes) is unfavorable.

Moreover, carbonyl stress is another route leading to cell damage that is associated with a number of human diseases [15]. It involves highly reactive dicarbonyl compounds that are formed mainly through non-enzymatic protein glycation and in turn are involved in the formation of various harmful cross-linked adducts, which are collectively called advanced glycation end-products (AGEs).

The bioactive properties of mushrooms are due to the polysaccharides, proteins, lipids and molecules from their secondary metabolism, such as terpenoids, eritadenine, ergothioneine and phenolic compounds [10,11,16,17,18]. The antioxidant activity of edible mushrooms was studied in mice and has been associated mainly with the polysaccharide fraction, ergothioneine, and phenolic compounds [9,10]. There is little information on the antiglycation activity of mushrooms. In a previous study, polysaccharides isolated from *Ganoderma lucidum* were supplemented in high-fat-diet/streptozotocin diabetic rats and found to decrease the level of AGEs and augment the activities of antioxidant enzymes [19].

In this study, *P. ostreatus* and *A. bisporus* were submitted to UV irradiation and the effects of the process on vitamin D_2_ content, as well as antioxidant and antiglycation properties were investigated to obtain overall knowledge regarding the impact of this process on the potential health benefit of mushrooms.

## 2. Materials and Methods

### 2.1. Chemicals

Chemicals were purchased from Sigma Aldrich (Milan, Italy).

### 2.2. Mushrooms

*P. ostreatus* and *A. bisporus* were purchased from the market. The irradiation treatment was performed as described previously [20]. In brief, about 10 kg of body fruits were used for each mushroom type, cut into 4 mm slices, spread on an oven rack in a single layer and air-dried at 37 °C for 48 h. During the first 24 h of air-drying, the mushrooms were irradiated using two fluorescent lamps installed at 30 cm above the mushroom layer. In accordance to most of the previous studies so far performed [8,12], a UV-B source was chosen for the irradiation. The lamps delivered a UV-B (280–315 nm) irradiance of 0.4 W/m^2^. At the end of the process, about 1 kg of dried mushroom was obtained from each mushroom type and the product was grinded to a fine powder using a Thermomix TM 31 (Vorwerk Contempora S.r.l., Milan, Italy). Control *P. ostreatus* and *A. bisporus* powders were obtained by processing mushrooms with the same drying and grinding procedure without UV irradiation.

### 2.3. Determination of Vitamin D_2_

Vitamin D_2_ and ergosterol were extracted in triplicate from non-irradiated and irradiated mushroom powders after saponification as described previously [21]. To check the recovery, preliminary samples of mushroom powder were added with 0.1 mL of vitamin D_3_ (400 mg/L in methanol). The observed recovery was always higher than 90%. Then, samples were analyzed without vitamin D_3_ in order to check the possible presence of vitamin D_2_ photoproducts with elution times close to that of vitamin D_3_ [22]. Vitamin D_2_ and ergosterol were identified by a previously published procedure [23]. A Shimadzu HPLC system (Kyoto, Japan), including an LC-20 AD pump and an SPD-M20A photodiode array detector operated by Labsolution Software, was used. The column was a C18 Sunfire (4.6 mm × 250 mm × 5 mm, Waters, Milan, Italy) and the mobile phase was methanol:water (95:5, *v*/*v*), at a flow rate of 1.0 mL/min. UV detection was performed at 254 nm. The retention times for vitamin D_2,_ vitamin D_3_ and ergosterol were 19.5, 20.5 and 23 min, respectively. The content of vitamin D_2,_ vitamin D_3_ and ergosterol were calculated on the basis of the calibration curve of pure compounds.

### 2.4. Recovery of Bioactive Fractions

Mushroom powders of *P. ostreatus* and *A. bisporus* were extracted in triplicate with cold water (0.25 g into 5 mL) for 24 h at 4 °C under magnetic stirring. Then, the mixtures were centrifuged (Centrikon T-42K, Kontron, Instruments, Milan, Italy) at 12,000 rpm for 30 min at 20 °C and the supernatant was used for further characterization [24]. The ethanol-insoluble fractions were obtained by adding 8.3 mL of 96% ethanol to 2 mL of the water extracts. The precipitate was recovered by centrifugation (Centrikon T-42K) at 12,000 rpm for 30 min at 20 °C and redissolved in water for further characterization [25]. The yield of water-soluble extracts and ethanol-insoluble precipitates was determined by measuring the weight of dry solids after drying in a vacuum oven at 70 °C and 50 Torr for 18 h.

### 2.5. Antioxidant Activity

The Folin–Ciocalteu (FC) assay was performed on the water-soluble fractions and the ethanol-insoluble fractions redissolved in water, as described previously [26]. A calibration curve was built using gallic acid. FC-reducing compounds were expressed as milligram of gallic acid equivalents (GAE) per gram of dry fraction.

The free radical scavenging capacity of the water-soluble fractions and the ethanol-insoluble fractions redissolved in water was evaluated using the stable 2,2-diphenyl-1-(2,4,6 trinitrophenyl) hydrazyl radical (DPPH) as described previously [27]. Additionally, 6-hydroxy-2,5,7,8-tetramethylchroman-2-carboxylic acid (Trolox) was used as a reference antioxidant and the results were expressed as micromoles of Trolox equivalents (TE) per gram of dry fractions.

The ferric ion-reducing antioxidant power (FRAP) assay was performed on of the water-soluble fractions and the ethanol-insoluble fractions redissolved in water as described previously [26]. FeSO_4_ was used for calibration and the results were expressed as micromoles of Fe II equivalents per gram of dry fractions.

### 2.6. Antiglycation Activity Using Bovine Serum Albumin (BSA)/Fructose Model Systems

The antiglycation activity was determined on the water-soluble fractions and the ethanol-insoluble fractions redissolved in water, as described previously [28]. The reaction mixture was prepared by adding 100 μL of sample extracts or standard diluted in water, 900 μL of 200 mM potassium phosphate buffer, pH 7.4 with 0.02% sodium azide, 300 μL of bovine serum albumin (BSA) solution (50 mg/mL of BSA in phosphate buffer) and 300 μL of fructose solution (1.25 M fructose in phosphate buffer). A BSA solution (blank sample) and control reaction without sample addition were prepared in parallel. The mixtures were incubated at 37 °C for 3 days in the dark and then analyzed for fluorescence on a Perkin-Elmer LS 55 Luminescence Spectrometer (Perkin-Elmer, Milan, Italy) with an excitation/emission wavelength pair of 350/420 nm and 5 nm slit width, read against phosphate buffer. Aminoguanidine was used as a positive control. For each sample extract, 3–4 dilutions were assessed in duplicate. Dose–response curves were built reporting % inhibition of BSA glycation as a function of sample or standard concentration. The results were expressed as milligrams of aminoguanidine equivalents (AG) per gram of dry fractions.

### 2.7. Statistical Analysis of Data

Experimental data were analyzed using one-way ANOVA with the least significant difference (LSD) as a multiple range test using Statgraphics 5.1 (STCC Inc., Rockville, MD, USA). These results are reported as the average ± standard error (SE).

## 3. Results and Discussion

### 3.1. The Effect of Mushroom Irradiation on Vitamin D_2_ Content

Initial content of vitamin D_2_ was low in both commercial mushrooms considered. This result is consistent with previous studies and could be associated with the conditions used for industrial cultivation of mushrooms that generally occurs with low exposure to sunlight [8,12]. UV irradiation led to an increase in vitamin D_2_ content from 3.1 to 37 µg/g d.w. and from 0.3 to 57 µg/g d.w. in *P. ostreatus* and *A. bisporus,* respectively (Table 1). The initial contents of ergosterol were 3600 and 5700 µg/g d.w. in *P. ostreatus* and *A. bisporus,* respectively (Table 1). These values fall in the range previously reported [29] and remained high after the generation of significant amounts of vitamin D_2_. This result may suggest that upon UV irradiation. the residual amount of ergosterol is still relevant for drug development and could contribute to the health-promoting effects of mushrooms [30].

**Figure 1:**
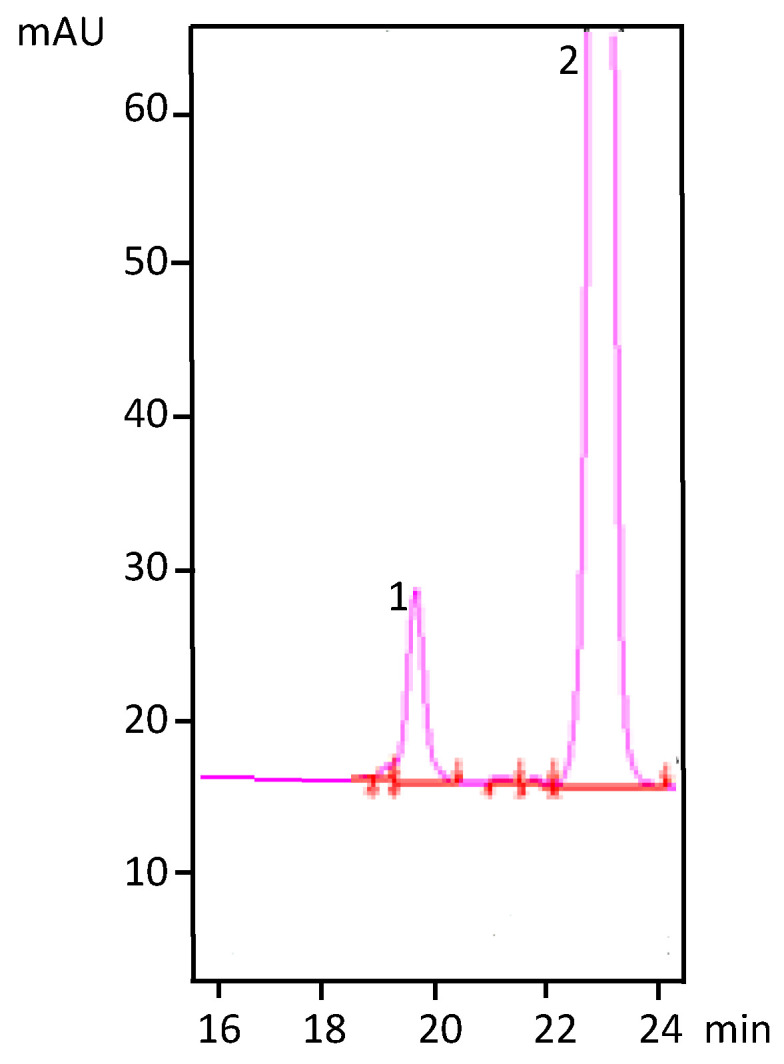
HPLC profile of the *n*-hexane extract of irradiated *P. ostreatus* powder. 1. Vitamin D_2_; 2. ergosterol.

The contents of vitamin D_2_ obtained were in the recovery range described by previous studies, i.e., from 3.9 to 741 µg/g d.w. for *A. bisporus* and from 27.89 to 239.67 µg/g d.w. for *P. ostreatus,* respectively [8]. In the present study, no vitamin D_2_ photoproducts were detected (Figure 1). According to [22], irradiation of *P. ostreatus* led to significant formation of vitamin D_2_, tachysterol_2_ and lumysterol_2_, but the conditions applied were different from those used in the present study and the amount of vitamin D_2_ produced was 141.2 mg/kg, i.e., higher than those observed in the present study.

The yield of vitamin D_2_ formation upon irradiation was previously found to depend on conditions that can be controlled during processing, such as the light source, the distance between the lamp and the mushroom, temperature and the duration of the irradiation. However, the yield also depends on factors that cannot be controlled, such as the shape of mushroom, which conditions the surface exposed to light and the moisture levels, that varies during drying [8]. The final powder obtained after irradiation can be standardized for vitamin D_2_ content by mixing the treated powder with untreated powder. Hence, considering a target final value for vitamin D_2_ of 40 µg/g d.w., a simulation was conducted to calculate the amount of irradiated powder that could be necessary to fortify vehicle foods in order to achieve health benefits (Table 2).

To perform this simulation, three studies were considered to define the most appropriate target foods and fortification levels, namely, a study in line with the regulation dealing with vitamin D fortification in the United States and Canada [31], a study considering the European regulation scenario on vitamin D fortification [32] and a study aimed at exploring food matrices for vitamin D fortification in low/lower-middle income countries [33]. These latter studies defined somewhat different fortification strategies due to different dietary habits and needs of the populations involved and to different regulations of the countries of interest. In any case, it can be observed that very low fortification levels of mushroom powder, in the range 0.03 to 0.38 g/100 g of food, could lead to the target fortification level, thus confirming the potential role of irradiation technology in increasing the intake of vitamin D (Table 2).

### 3.2. The Effect of Mushroom Irradiation on Antioxidant Activity

Considering the variety of mushroom compounds that can act as antioxidants, various extraction procedures have been proposed to study the antioxidant activity of mushrooms. Comparing the yields of ethanol and hot water extractions for various mushroom species, it was observed that ethanol only accounted for 5.89–18.89% of solids, while hot water fraction accounted for 38.3–57.2% of total solids [34]. Moreover, information obtained by using hot water is considered more valuable because it corresponds to the procedure used to recover bioactive compounds from medicinal mushrooms by Chinese traditional medicine [34]. However, studies conducted on *P. citrinopileatus* have shown a greater bioactivity in vitro of the cold aqueous extract compared to the hot water extract due to protein denaturation and degradation of phenolic compounds at high temperatures [24]. Moreover, the ethanol precipitation of the aqueous extract has been proposed for the isolation of proteoglycans with anticancer properties [25]. Hence, in the current study, both the cold water extract and ethanol precipitate fractions of *P. ostreatus* and *A. bisporus* were studied to assess the effects of UV irradiation. The water-soluble fraction of *P. ostreatus* accounted for 54% of solids, while the ethanol-insoluble fraction was 7.2% of solids (Table 3). Higher percentage of the water extracts was observed for *A. bisporus*, i.e., 69%, but the percentage of ethanol-insoluble fraction was lower, i.e., 3.3% (Table 3). Due to the high extraction yield, the cold water extracts can be considered representative of the mushroom matrix to investigate the effect of irradiation on potential bioactive components.

The antioxidant activity in both non-irradiated and UV-irradiated mushrooms was evaluated by the FC, FRAP and DPPH assays (Table 3).

The levels of FC-reducing compounds for the aqueous extracts of *P. ostreatus* and *A. bisporus* before irradiation were 24.6 and 16.2 mg GAE/g of fraction, respectively. These values can be considered to be high according to the ranking defined previously in a screening study on 23 species of mushrooms, which reported levels of FC-reducing compounds of the aqueous extract in the range of from 2 to 37 mg GAE/g [35]. The ethanol-insoluble fractions of both mushrooms showed a higher specific content of FC-reducing compounds than those of the respective water-soluble fraction for both mushrooms. This result may suggest that bioactive compounds partitioned mostly into the ethanol-insoluble fraction. UV irradiation decreased the FC-reducing compounds of *P. ostreatus* by 20%, while the decrease was lower for *A. bisporus* (7%).

The DPPH values for the water extracts of *P. ostreatus* and *A. bisporus* before irradiation were 56 and 48 µmol TE/g fraction. As observed for the FC-reducing compounds, the specific DPPH values for the ethanol-insoluble fractions of both mushrooms were higher than those of the corresponding water-soluble fractions. The genus *Agaricus* has been previously found to have the highest DPPH radical scavenging properties among the most widely appreciated cultivated mushrooms [36]. UV irradiation decreased the DPPH values of all fractions, except for the ethanol-insoluble fraction of *A. bisporus*.

The FRAP values for the water extracts of *P. ostreatus* and *A. bisporus* before irradiation were 82 and 208 µmol FeII/g fraction, respectively. As observed for FC and DPPH, higher specific FRAP values were present in the ethanol-insoluble fractions than in the water-soluble fractions for both mushroom species, with values of 222 and 473 µmol FeII/g fraction. This result may be related to the preferential partition of the bioactive compounds in the ethanol-insoluble fractions. A screening of 1943 plant-based foods revealed that the average FRAP value is 115.7 µmol FeII/g [37]. Hence, mushrooms can be considered important as a dietary source of reducing compounds. On the other hand, irradiation caused a marked decrease in the FRAP values, except for the ethanol-insoluble fraction of *A. bisporus*.

### 3.3. The Effect of Mushroom Irradiation on Antiglycation Activity

The antiglycation activity in both non-irradiated and UV-irradiated mushrooms was evaluated by the BSA/fructose assay (Table 4).

The water extracts of *P. ostreatus* and *A. bisporus* were able to inhibit protein glycation, exhibiting 113 and 83 mg AG/g fraction. As observed for the antioxidant activity, which was higher in the ethanol-insoluble fractions, higher levels of antiglycation activity was observed in the ethanol-insoluble fractions with respect to the water-soluble fractions, corresponding to 177 and 555 mg AG/g fraction for *P. ostreatus* and *A. bisporus*, respectively. There is no information on the antiglycation activity of these mushroom species. In previous studies, the antiglycation activities of a crude water-soluble fraction extracted from the sclerotia of *Inonotus obliquus* [38] and *Lignosus rhinocerus* [39] were found to be 23 and 133 mg AG/g fraction. Compared to these latter studies, the antiglycation activities of the ethanol-insoluble fractions of *P. ostreatus* and *A. bisporus* were found to be much higher. However, even higher antiglycation activity was observed for a polysaccharide purified from the fruiting body of *Boletus snicus*, which was found to have the same antiglycation activity of aminoguanidine in the BSA/glucose model system, i.e., 1000 mg AG/g [40].

The UV irradiation process did not cause any significant change to the antiglycation activity of *P. ostreatus* and *A. bisporus* (Table 4). Despite redox-active compounds generally possessing antiglycation activity, this activity is also dependent on metal chelation and carbonyl-trapping abilities [16].

## 4. Conclusions

*P. ostreatus* and *A. bisporus*, which are among the most cultivated mushroom species in the world, were able generate relevant amounts of vitamin D_2_ through UV irradiation. The concept that this latter technology can address the global need of vitamin D_2_ was highlighted by providing a scenario of possible future application of the vitamin D_2_ enriched mushroom in target foods. *P. ostreatus* and *A. bisporus* were also found to possess high antioxidant and antiglycation activities. Irradiation caused a decrease in antioxidant activity in both mushroom species but did not affect antiglycation activity. The ethanol-insoluble fraction of *A. bisporus* was not affected by UV irradiation. Results from this research open up a new area of investigation. In fact, while the relevance of UV irradiation of mushrooms to address the global need of vitamin D_2_ has attracted a lot of research interest, the identification of the effects of irradiation on the mushroom matrices is still lacking and deserves particular attention.

One limitation of the current study is that it included only two mushroom species. Considering the wide biodiversity among mushrooms, future studies should be directed to extend knowledge on the effects of irradiation to other mushroom species in order to assist a more efficient design of the process for the generation of vitamin D_2_ with a major focus on the retention of bioactive properties.

## Figures and Tables

**Table 1:** Vitamin D_2_ and ergosterol content in non-irradiated and UV-irradiated mushrooms ^a^.

Mushroom	Vitamin D_2_ μg/g d.w	Ergosterol μg/g d.w.
*P. ostreatus*	3.1 ^c^ ± 0.1	3600 ^c^ ± 400
*P. ostreatus*_UV_	37 ^b^ ± 0.1	3200 ^c^ ± 300
*A. bisporus*	0.31 ^d^ ± 0.1	5700 ^a^ ± 300
*A. bisporus*_UV_	57 ^a^ ± 0.1	4800 ^b^ ± 300

^a^ Values are average ± SE. Different letters in the same column (a–d) indicate significant differences among samples (LSD, *p* < 0.05).

**Table 2:** Modelled formulations of foods with irradiated mushroom powder to achieve a target level of vitamin D_2_^a^.

Food Category	Target Level for Vitamin D μg/100 g Food	Reference	Amount of UV-Irradiated Mushroom Powder g/100 Food
Ready-to-eat breakfast cereals	8.75	[31]	0.22
Milk	1.05	[31]	0.03
Yogurt	2.225	[31]	0.06
Margarine	8.275	[31]	0.21
Edible oil	15	[32]	0.38
Milk	2	[32]	0.05
Wheat flour	2.8	[32]	0.07
Milk	1	[33]	0.03
Orange juice	10.5	[33]	0.26

^a^ The mushroom powder considered was obtained from *P. ostreatus* and *A. bisporus* through UV irradiation as described in the Material and Methods Section and contained 40 µg/g of vitamin D_2_.

**Table 3:** Fraction yields and antioxidant activity evaluated by the FC, DPPH and FRAP assays of the water extracts and ethanol-insoluble fractions of *P. ostreatus* and *A. bisporus* before and after UV irradiation.^a^.

Mushroom and Treatment	Fraction Yield g/100 g	Antioxidant Activity		
		FC mg GAE/g fraction	DPPH µmol TE/g fraction	FRAP µmol FeII/g fraction
*P. ostreatus* WE	54 ^b^ ± 3	24.6 ^c^ ± 1.3	56 ^c^ ± 4	82 ^d^ ± 5
*P. ostreatus*_UV_ WE	54 ^b^ ± 3	19.6 ^d^ ± 1.5 (20%)	48 ^d^ ± 1 (14%)	58 ^e^ ± 14 (29%)
*A. bisporus* WE	67 ^a^ ± 1	16.2 ^e^ ± 0.5	48 ^d^ ± 6	208 ^b^ ± 7
*A. bisporus*_UV_ WE	70 ^a^ ± 1	15.0 ^f^ ± 0.2 (7%)	37 ^e^ ± 7 (23%)	131 ^c^ ± 28 (37%)
*P. ostreatus* EP	7.2 ^c^ ± 0.1	32.1 ^b^ ± 0.6	71 ^b^ ± 1	222 ^b^ ± 1
*P. ostreatus*_UV_ EP	7.3 ^c^ ± 0.1	25.8 ^c^ ± 0.7 (20%)	49 ^d^ ± 3 (31%)	127 ^c^ ± 14 (43%)
*A. bisporus* EP	3.4 ^d^ ± 0.1	53.4 ^a^ ± 1.6	119 ^a^ ± 14	473 ^a^ ± 43
*A. bisporus*_UV_ EP	3.2 ^d^ ± 0.1	53.4 ^a^ ± 0.8	103 ^a^ ± 17	505 ^a^ ± 14

^a^Values are average ± SE; values in brackets are the percent decreases for the UV irradiated samples with respect to non-irradiated samples. WE = water extract, EP = ethanol precipitate, FC = Folin–Ciocalteu, DPPH = 2,2-diphenyl-1-(2,4,6 trinitrophenyl)hydrazyl radical, FRAP = ferric ion-reducing antioxidant power, GAE = gallic acid equivalents, TE = Trolox equivalents. Different letters in the same column (a–e) indicate significant differences among samples (LSD, *p* < 0.05).

**Table 4:** Antiglycation activity of the water extracts and ethanol-insoluble fractions of *P. ostreatus* and *A. bisporus* before and after UV irradiation.

Mushroom and Treatment	Antiglycation Agents mg AG/g fraction
*P. ostreatus* WE	113 ^c^ ± 22
*P. ostreatus*_UV_ WE	131 ^c^ ± 25
*A. bisporus* WE	83 ^d^ ± 9
*A. bisporus*_UV_ WE	89 ^d^ ± 10
*P. ostreatus* EP	177 ^b^ ± 10
*P. ostreatus*_UV_ EP	170 ^b^ ± 6
*A. bisporus* EP	555 ^a^ ± 80
*A. bisporus*_UV_ EP	500 ^a^ ± 89

Values are average ± SE. WE = water extract, EP = ethanol precipitate, AG = aminoguanidine. Fraction yield is reported in Table 3. Different letters in the same column (a–d) indicate significant differences among samples (LSD, *p* < 0.05).
